# The nature and significance of social ontology

**DOI:** 10.1007/s11229-023-04142-1

**Published:** 2023-04-12

**Authors:** Francesco Guala, Frank Hindriks

**Affiliations:** 1 Department of Philosophy, University of Milan, Via Festa del Perdono 7, Milan, Italy; 2 University of Groningen, Groningen, Netherlands

**Keywords:** Consilience, Ecumenism, Fallibilism, Meta-ontology, Social ontology, Unification

## Abstract

We propose a bridge-builder perspective on social ontology. Our point of departure is that an important task of philosophy is to provide the bigger picture. To this end, it should investigate folk views and determine whether and how they can be preserved once scrutinized from the perspective of the sciences. However, the sciences typically present us with a fragmented picture of reality. Thus, an important intermediate step is to integrate the most promising social scientific theories with one another. In addition to this, social ontology can provide input to and benefit from other philosophical disciplines that engage in normative theorizing. Thus, we propose that social ontology connects not only with folk ontology and scientific ontology but also with fields such as ethics and political philosophy. Building bridges between them serves to formulate a credible and encompassing worldview that is of theoretical and practical significance.

Meta-ontology is commonly conceived of in fairly narrow terms. According to Peter van Inwagen, who coined the term, *the* meta-ontological question is: ‘What are we asking when we ask ‘What is there?’ (1998, 233) We understand it more broadly in terms of questions such as: ‘Why do ontology?’ And ‘How can or should ontological questions be answered?’ Questions such as these concern the motivation, method, purpose and significance of, in our case, social ontology. Thus, our main question is:

(Q1) What is the task of social ontology?

Social ontology should clarify what the social world is made of and how social entities are related to non-social ones, including psychological and physical entities. However, it should also reflect on the extent to which different images of reality – those provided by various branches of science as well as those that inform our everyday activities – are compatible. In Wilfred Sellars’ (1963) terms, it should in part be concerned with how the manifest image of the social world relates to the scientific image. In light of this, we ask:

(Q2) How does social ontology relate to folk ontology and scientific ontology?

We argue that social ontologists should use both folk intuitions and scientific findings as relevant sources of knowledge. Thus, we defend an *ecumenical* conception of social ontology.

To explain our view and clarify what motivates it, we contrast it in Sect. 1 with three non-ecumenical or monist options. The first option is that social ontology is a purely philosophical discipline. The second option equates it with scientific ontology, the third with folk ontology. Against monism, we argue that it is a respectable goal of ontology to provide ‘the bigger picture’. More specifically, it is important that philosophers achieve consilience between scientific ontology and folk ontology. 1

At the end of that section, we ask how consilience is to be achieved. We point out that the social sciences are fragmented. But explaining ‘how it all hangs together’ requires a coherent ontology. Thus, we advocate integrating or unifying social scientific theories. This serves to develop a better overall understanding of the world we live in. Because unification and consilience play a central role in it, we conceive of our ecumenical proposal as ‘a bridge-builder conception’ of social ontology.

Along the way, we discuss existing alternatives. We focus on three kinds of monism that are not just ideal types, but that are actually defended within social ontology. We also discuss other forms of ‘pluralism’, which typically combine the three sources in a more pragmatic manner. In Sect. 2 we illustrate our ecumenism using some of our own work, on the assumption that we have practiced what we now preach all along. Subsequently, we ask how social ontology can contribute beyond its own boundaries. Thus, our third and final question is:

(Q3) What is the broader significance of social ontology?

In Sect. 3, we argue that social ontology is important for normative theorizing. It is relevant to disciplines such as ethics, the philosophy of gender and race, the philosophy of law and political philosophy. Thus, our ecumenical conception does not only capture the nature, but also the significance of social ontology.^2^

## Monism vs. ecumenism

Ecumenism, we propose, is the view that social ontology is best practiced by integrating two sources of knowledge: science and common sense. It is ecumenical in that it takes both sources seriously and promotes unity among them.^3^ This implies that there is no sharp divide between social ontology as a philosophical discipline, on the one hand, and scientific ontology and folk ontology on the other. It also means that folk views do not necessarily have to give way to science, but that they can often be preserved.

### Folkism, scientism and analyticism

According to monism, social ontology needs to consider only one source of knowledge. Hence, there are three kinds of monism. The first two identify social ontology respectively with folk ontology and scientific ontology. For lack of better terms, we label them ‘folkism’ and ‘scientism’. According to the third one, ontology is a strictly philosophical discipline, which is practiced from the armchair. As it often overlaps with ‘analytic metaphysics’, we refer to it as ‘analyticism.’

Both folkism and scientism issue in *a posteriori* knowledge, while in its pure form, analyticism regards ontology as an *a priori* inquiry. Furthermore, analyticism attributes special and unique expertise to philosophers, and scientism to scientists. According to folkism, no special expertise is needed to have knowledge about social ontology. Finally, in folkism and scientism, philosophy plays an ancillary role, as an ‘underlaborer’. In contrast, analyticism attributes a special domain to it along with a distinct method.

These three kinds of monism serve as foils that are useful for clarifying what ecumenism is. They are extreme views, and because of this, one might dismiss them as distracting distortions. We like to think of them instead as insightful ideal types. Following Sellars’ (1963) precepts, we discuss them as such, adding more detail as we go along (see also O’Shea, 2007, 12–13). However, we do not want the ideal types to be dismissed as straw men. Hence, we first show that each of these views, or something near enough, is in fact defended within social ontology.

Margaret Gilbert regards as her ‘primary aim … to make explicit the structure of certain everyday concepts’ (1989, 3). This suggests that she subscribes to folkism. In contrast, Alexander Rosenberg argues that we should not take common sense seriously at all, and explicitly distances himself from those who seek ‘to reconcile science with common sense or the manifest image or the wisdom of our culture’ (2014, 17). Rosenberg, in fact, supports scientism.^4^ Finally, Kit Fine consciously pursues analytic metaphysics. He writes that ‘nothing […] requires one to take [the world described by] science as opposed to our common sense world as real’ (2017: 111), and that metaphysics ‘is distinguished, in part, from physics and other branches of science by the a priori character of its methods’ (2012: 9). Recently, he has used this methodology to propose an ontology of social groups (Fine, 2020).^5^

The upshot is that, even though the three views are ideal types, there are clear resemblances between them and positions that are actually defended within social ontology. In what follows, we critically discuss analyticism, folkism and scientism. In Sect. 2, we show how insights from different sources can be integrated: social ontology needs inputs both from the social sciences and from common sense. Furthermore, we argue that integrating the two is the job of philosophers.

### From monism to consilience

Proponents of monism believe that one source of knowledge suffices for doing social ontology. In this section, we argue that social ontology is not an a priori inquiry and that it needs input from the sciences. Then we explain why folk ontology deserves to be taken seriously as well. To this end, we critically discuss the three kinds of monism. We start from the ideal-types introduced above, refining them as we go along where needed. Our ultimate goal is to explain why we defend ecumenism. To keep the analysis as brief and clear as possible, we discuss the views in relation to ontology in general. In Sect. 2, we bring the fruits of this discussion to bear on social ontology.

According to analyticism, philosophers can acquire knowledge about ontology by reflecting on concepts. But how can this *a priori* method generate knowledge about what there is? It could do so if our concepts accurately represented the world. But this, we propose, cannot simply be assumed. So, it seems that the best that philosophers can hope for is that concepts provide us with knowledge *if they are adequate*.^6^ And this is at least in part an empirical question. Without external input, ontology is destined to be a sterile enterprise.

In defense of analyticism, it could be said that philosophers have special expertise – they are ‘competent users’ of the relevant concepts. Another way to put it is to say that lay people have untutored intuitions, while philosophers have sophisticated intuitions that can be used to refine the concepts at issue. In this vein, Steven Hales argues that ‘the modal intuitions of professional philosophers are much more reliable than either those of inexperienced students or the “folk”’ (Hales, 2006, 171).^7^ If correct, the quality of the output of philosophical reflection will surpass that of folk views. But will a priori reflection tell us what there is?

Perhaps ontology concerns the *necessary* features of what there is, such as how some entities depend on others.^8^ That some general aspects of dependence relations are accessible by armchair reflection is a popular idea among contemporary metaphysicians. That dependencies among specific classes of entities or properties are discoverable this way, however, is much more controversial. Is collective acceptance a *necessary condition* for the existence of institutions? Is money *essentially* a store of value? There is no reason to believe that philosophers have privileged insights into issues of this kind.

A division of labor seems more plausible. While philosophers may have something to say about what causation or dependence are in general, scientists typically tell us what causes what or what depends on what. Some philosophers in fact regard philosophical knowledge as continuous with science (Jackson, 1998; Williamson, 2007). This means that not all knowledge about ontology is *a priori.* And this in turn explains how it can extend beyond concepts to what there is. If at least some knowledge about ontology is *a posteriori*, then there seems to be ample reason to welcome input from the sciences. Now, we grant that philosophers have special expertise when it comes to topics such as causal relations and dependence relations. What we resist is the idea that scientific insights are irrelevant to these topics.

Proponents of scientism reject the idea that common sense or philosophy might be legitimate sources of knowledge for social ontology. Insofar as common sense is concerned, the idea is that scientific knowledge supersedes folk beliefs. Because of this, we can do without the latter. Now, perhaps science can do without common sense, but philosophy cannot. To gain a better understanding of the world we inhabit, it is important to us to know to which extent common sense can be preserved. Rosenberg is not entirely wrong when he claims that ‘science forces on us a very disillusioned “take” on reality’ (2014, 17). As we see it, this provides reason to explore whether it offers us the whole story.

The sciences provide at best a partial perspective on what there is. As J.L. Austin (1962, 8) pointed out, folk ontology is committed to the existence of so-called middle-sized dry goods, such as tables and chairs as well as books and pictures (strikingly, he also took them to include rivers and rainbows). To add some examples of institutional objects, we may also include gavels, police uniforms and wedding rings. Now, objects such as tables and gavels do not figure in physical theories, while atoms in a void do. But this does not necessarily entail that there are no tables or gavels, as it appears to us from an ordinary perspective.

Wilfrid Sellars (1963) distinguished between the manifest image and the scientific image. This distinction runs roughly parallel to that between folk ontology and scientific ontology. Sellars argued that each of them is partial. Because of this, they are to be combined in order to arrive at a more complete account of the way the world is. Scientism carries the risk that many interesting and important entities are left out, simply because they are not mentioned by current scientific theories. Social ontology can prevent this by taking seriously the world in which people think they live.

Few if any social scientific theories feature objects such as gavels, police uniforms or wedding rings.^9^ For instance, a game theoretic model with police officers and criminals as players features their preferences and expectations along with the rules of the game, none of which are likely to even mention police uniforms, let alone provide an account of them. This confirms Sellars’ claim that the sciences offer only a partial perspective on what there is. Yet, gavels, uniforms, and rings play important roles in everyday life.

By including them, we can tell ‘the whole story’ that we are after, which preserves common sense inasmuch as possible. Thus, we believe that a respectable goal of ontology is to provide ‘the bigger picture’ or, in terms of another metaphor, to explain ‘how it all hangs together’. In other words, our aim is to formulate a coherent and comprehensive theory by using both common sense and science as sources. What we call ‘consilience’ is the intended result of this endeavor. Thus, the overarching goal of our ecumenical method is to achieve consilience.^10^

### Against folk infallibilism

Some monist approaches have doubtful epistemic credentials. There is little or no reason to take folk beliefs about astrophysics seriously: it turns out, for instance, that what goes up need not come down. This is why there is ample reason to expect scientific physics to supersede folk physics in many respects. So, folk physics has low credence, while scientific physics has high credence.^11^

Strikingly, the situation seems to be reversed when it comes to social ontology. First, a substantial number of philosophers believe that, by and large, folk ontology must be correct, because social reality is mind-dependent: it is constituted by what human beings think, decide and do. This is taken to imply that they have privileged access to beliefs that provide unfailing information. Such folk infallibilism has been defended with respect to institutions. The idea is that institutions depend on rules, and that these rules are in force because people accept them. Because of this, they cannot be mistaken about the contents of these rules (Ruben, 1989; Thomasson, 2003).^12^

In certain cases, this is rather plausible. Consider a drinking norm according to which students at certain colleges are expected to engage in excessive drinking on the weekends. There is little reason to think that they are mistaken about the existence of this norm. Yet, as it turns out, actual support for the practice is rather low, as many dislike hangovers more than they like drinking. The practice of excessive drinking is kept in place only by the thought that others accept the norm, and the peer pressure that students exert on each other because of this. This phenomenon, known as ‘pluralistic ignorance’ (Prentice & Miller, 1993), demonstrates that infallibilism has limited scope of application, for the existence of social entities, practices, and institutions is compatible with massive ignorance about the mechanisms that keep them in existence.

In other cases, there is reason to doubt that people even know the contents of the rules that constitute the relevant social entities. For instance, the features of paper money are very complex so as to prevent or mitigate forgery. You will know a few salient features, but nowhere near all of them. Few are familiar with the fine print, the watermark, or the composition of the paper. As another example, there are laws that even experts might not be aware of. For instance, it is a legal requirement in Milan to smile at all times (except in hospitals and at funerals).^13^ Such weird laws, which are no longer enforced if they ever were, are often long-forgotten. This is possible because laws are accepted as systems (Hart, 1961). Hence, the fact that the social is mind-dependent does not imply folk infallibility.

A second factor in favor of folkism is that many philosophers have a low opinion about the track record of the social sciences. Katherine Hawley (2018) has recently argued that, because of this, social scientific theories are basically useless for social ontology. Her point of departure is the view that scientific ontology proceeds by means of inference to the best explanation. Roughly, the idea is that those entities exist that are postulated by our best scientific theories. But this only works if those theories are good enough. And social scientific theories do not meet this bar.

How high is this bar? Inspired by Psillos (1999), Hawley (2018, 189) requires that the relevant science is mature ‘in the Kuhnian sense’, and makes successful novel predictions. Now, in particular economists and psychologists work within pretty well-defined paradigms, such as rational choice theory and social identity theory, and generate a substantial amount of empirical studies within their confines. So, what they do looks a lot like normal science in Kuhn’s sense. Social science, moreover, is deeply involved in devising policies and making predictions upon which both individual citizens and institutions rely for planning purposes. There is an obvious sense in which a solid backbone of reliable, projectible knowledge is essential for the existence of any complex society.

Hawley admits candidly that she ‘cannot possibly evaluate the historical state of development and levels of empirical success of the various social sciences—this would be a mammoth task for which I am completely unqualified’ (2018, 190). But this does not stop her from stating that ‘even at the most superficial level a number of phenomena indicate that the prospects for securely basing social metaphysics via inference to the best explanation from social science are currently faint’ (2018, 190). We believe that this conclusion is superficial and premature.

To be sure, it would be foolish to give the green light indiscriminately to all fashionable theories. But there are vast areas of the social sciences with a record of explanatory and predictive success that is at least as good as that of equally vast and important areas of, say, bio-medical science. The controversies and limited successes displayed by virology during the recent Covid-19 pandemics should provide philosophers with food for thought: presumably we should *not* infer from that messy story that viruses do not exist.

The right conclusion is that both folk ontology and scientific ontology should be regarded as fallible. Earlier we argued that both are respectable sources of input for social ontology. Here the point is that no source of information is perfectly reliable, and that we must always consider the possibility of error. This obviously allows for the possibility that scientific findings can supersede folk beliefs. But it also means that certain folk beliefs might be on a rather secure footing and that philosophers should not give them up easily.

To sum up, we do not want to claim that the epistemic credentials of the social sciences are particularly high. But someone who applies the ecumenical method should have a good enough sense of them to be able to select the best theories about the topic at issue. And those theories deserve to be taken seriously. We are certainly better off with them than without them.

### Dualism and pluralism

We have used the ideal-types to defend ecumenism along with fallibilism. We hope that this has clarified our position and its motivation. But is there anything new about it? Many social ontologists are dualists in the sense that they take philosophy seriously as a source of knowledge along with either folk ontology or scientific ontology. Lynne Rudder Baker’s (2007) *The Metaphysics of Everyday Life* is an example of folk dualism in this sense.^14^ Harold Kincaid may be considered a scientific dualist, as is suggested by his claim that ‘conceptual metaphysics without close ties to science is of minimal value’ (2021, 41).^15^

However, even if others practice what we preach, this is not an embarrassment. Our main motivation for this paper is to contribute to and stimulate a debate about meta-ontology: now that the discipline is coming of age, it is important to be more self-conscious about the methodology of social ontology. In fact, we suspect that some of our fellow travelers might be what we call ‘pragmatic pluralists’, instead of ecumenists. They believe that each of the three sources of knowledge might be useful to social ontology at some point or other, and make use of them in a non-principled manner. Our ecumenical approach is more ambitious. As we see it, consilience is a central goal of social ontology. Hence, its success depends on philosophers’ capacity to combine folk ontology with scientific ontology. This goal distinguishes our approach from pragmatic pluralism.

At this point, we are in a position to answer Q2: How does social ontology relate to folk ontology and scientific ontology? We claim that social ontology is *ecumenical* in that it takes philosophy, folk ontology and scientific ontology seriously, and promotes unity among them.^16^

### Unification

Our endeavor of achieving consilience is similar to Sellars’ project of constructing a ‘synoptic’ view of the world that saves both the manifest and the scientific images insofar as possible. This requires explicating the ontological commitments implicit in folk and scientific theories and combining them. Sellars appreciated that doing so is difficult. For one thing, the relation between the theoretical constructs of science and the manifest image is far from straightforward. Due to conflicts, they cannot both be saved in their entirety. Thus, combining them requires resolving inconsistencies between the images.

Furthermore, Sellars understood – unlike some of his contemporaries – that the scientific image is not a single unified picture but a plurality of representations that overlap only in part. The sciences do not deliver a unified, transparent, consistent representation of the world. Instead, they present us with a fragmented or dappled picture of reality.^17^ Because of this, social ontology requires a non-trivial effort directed at clarifying, interpreting, sometimes qualifying the existential claims that are implicitly or explicitly made by the various branches of social science. It also requires resolving inconsistencies within the scientific image.

To this end, our best social scientific theories need to be integrated or unified.^18^ Michael Friedman (1974) proposed a deductive account of theoretical unification on which it is a matter of deriving the laws of one theory from those of another one. Philip Kitcher (1981) maintained that a theory becomes more unified by applying its core argument to a wider variety of phenomena. Both believed that explanation is achieved by means of unification. But many of these ideas have now lost their appeal. Laws play at best a marginal role in our conception of theories. And explanation is now commonly explicated in terms of causal relations, such as tendencies or invariances (Cartwright, 1989; Woodward, 2005).

On a causal account, explanation does not require theoretical unification. What it takes instead is a theory or model that adequately captures a causal relation. Now, a theory can be modified so as to explain a wider range of phenomena. And it can come to do so in terms of fewer explanatory factors. Both are ways to increase the explanatory efficiency or explanatory power of a theory, or ways of achieving *explanatory* unification. However, if these modifications occur within a theory, they do not involve *theoretical* unification. Although theoretical unification can be a means to explanatory unification, they are distinct (Mäki, 2001; Hindriks, 2022b).

Theoretical unification has a fair share of critics. One worry is that unified theories are difficult to confirm, because they are so coarse-grained (Cartwright, 1999; Morrison, 2007; but see Mäki and Marchionni 2009). Others believe that an adequate scientific understanding of specific phenomena is best achieved through local theories and models (Aydinonat & Ylikoski, 2018). Such philosophers celebrate theoretical diversity claiming that multiple incoherent scientific models are more useful for explanation or prediction than a unified theory. Now, we do not claim that unification is always a virtue. And we could even grant that, for scientific purposes, it may be undesirable. Our point is instead that some explanatory questions may remain unanswered once the job of science is done. It may be unclear, for example, how different domains or aspects of reality are related. This is why unification is a prerequisite *for ontology*. For this purpose, the worries just mentioned are simply irrelevant.^19^

Another worry is that unified theories are too abstract to be informative. This is not so easily dismissed. But the question is: informative in which respect? If consilience is the goal, a unified theory is to reveal ‘how it all hangs together’. And if it does so, it need not be a problem if the level of detail it provides about a particular phenomenon is less than it could have been. Furthermore, it is far from obvious that a unified theory will be less informative. If successful, a theoretical unification leads to an increase in explanatory power. Hence, it will be more informative in the sense of filling the remaining gaps (if any) between domain-specific models and explanations. Ultimately, the proof of the pudding is in the eating. We submit that the unified theory that we present in Sect. 2 is not only informative for its purpose, but also more informative than its constituents taken independently.

Given a causal account, theoretical unification is not a matter of deduction. Instead, it is a serendipitous process in which several theories are used to reconceptualize causal relations. To make this work, they have to be adjusted so as to achieve the requisite fit. Hence, social ontology cannot simply be considered an underlaborer of science. Philosophers ought to assess, criticize, clarify, rather than merely accept a body of knowledge that has been produced elsewhere.

In sum, an important aim of social ontology is consilience, to provide a coherent picture of the social world. Because of this, folk ontology and scientific ontology should both serve as inputs to social ontology (Q2). Furthermore, achieving consilience requires unification (Q1). This provides answers to the first two questions we started with. In Sect. 3, we address the broader significance of social ontology (Q3). Before then, we discuss our own work to illustrate the methodology of social ontology that we have just defended.

## Social ontology in action

At this point, it is useful to examine a concrete example of ecumenist social ontology. In our own work, we have addressed topics such as social practices and institutions, conventions and social norms, regulative and constitutive rules, as well as social objects, their constitution and their functions. Here we will focus on institutions and institutional objects, with the aim of illustrating the main points outlined earlier.

### Equilibria and rules

The two most influential schools of thought about social institutions or structures conceive of them either as equilibria or rules. In the jargon of contemporary social science, an *equilibrium* is a set of individual strategies such that no individual has an incentive to change their course of action unilaterally. If individuals encounter a situation repeatedly, it issues in a behavioral regularity. According to the equilibrium approach, such a regularity is a social structure. The oldest and most famous example of an equilibrium theory is David Lewis’ (1969) theory of social conventions. Lewis argued that conventions are equilibria in coordination games and thereby solutions to coordination problems. Examples include words in a language and driving on a particular side of the road. His theory is so elegant and simple that some theorists have tried to use coordination equilibria as the basis for an ontology of institutions more generally (e.g. Schotter, 1981, Calvert, 1995).

*Rule* theories are quite varied. Some focus on the normative dimension of institutions. The basic idea is that rules prescribe certain behaviors. Furthermore, the very fact that it is in force is a reason to conform to it, or at least that people regard it as such (Wittgenstein, 1953; Hart, 1961; Bloor, 1997; Brennan et al., 2013). According to a related strand of thought, rules give meaning to actions performed within the context of a social structure. Furthermore, they confer significance on the objects that are used in that context (Giddens, 1984; Sewell, 1992). A popular variant of this idea is that, if constitutive rules are in force, it is possible to describe certain actions in institutional terms, while the same actions would have little or no significance independently of those rules (Rawls, 1955; Searle, 1995; Tuomela, 2013).

Equilibrium theories and rule theories seem to have little in common. To be sure, the behaviors of people who conform to a rule will form a pattern. Yet, the strength of equilibrium theories resides in how they explain such patterns. The underlying idea is that people have expectations about what other people do and that, given those expectations, they will prefer certain actions over others. In Cristina Bicchieri’s (2006) terms, their preferences will be conditional. For instance, if a situation constitutes a coordination game, they will want to adapt their behavior to whatever is customary, given that everybody benefits from converging on a particular pattern, such as using the same words for the same things. While equilibrium theories excel at explicating the behavioral dimension of social structures, rule theories capture their normative dimension as well as their semantic or symbolic dimension.^20^

In past and ongoing work, we have proposed that rules and equilibria are the two building blocks of social structures. If we are right, theories that invoke only one of these notions are bound to be partial. They capture only one or two of their dimensions instead of all three. But how can they be combined? On the face of it, they are worlds apart. In spite of this, we have proposed *two* kinds of unified theories, one for social structures that facilitate coordination and one for social structures that enable cooperation.

Lewis argued that, in coordination games, people will want to do what others do. So, he invoked precedence to explain the continued existence of conventions. But what explains the emergence of a regularity in the first place? It can be explained in terms of correlating or signaling devices, such as traffic lights and wedding rings. They help people to converge on a particular solution to a coordination problem. However, signaling devices are not self-interpreting. In light of this, we propose that they feature in signaling rules, which confer meaning on those devices. The basic structure of such a rule is (with ‘*A*’ for an action and ‘*D*’ for a signal): If *D*, do *A.*^21^ As those rules specify the strategies according to which people act, we conclude that effective signaling rules will be in equilibrium, which is why we call our theory ‘the Rules-in-Equilibrium Theory’ (Guala & Hindriks, 2015, Hindriks and Guala 2015, Guala, 2016).

More recently, one of us has proposed ‘the Rules-*and*-Equilibria Theory’ (Hindriks, 2019, 2021, 2022a). Instead of coordination games, it pertains to mixed-motive games and cooperation problems, such as public goods games or the famous Prisoner’s Dilemma game. To solve such problems, people have to somehow overcome the conflicts of interest that exist between them. Suppose a farmer helps his neighbor to bring in the harvest. In this case, the question is why the latter would return the favor. Rules play a vital role in this respect as well. Instead of their symbolic dimension, it is their perceived normative dimension that is relevant here.

Rules can constitute social norms and people might have or see reason to follow them. Now, equilibrium theories reduce norms to sanctions, which change the benefits or payoffs people incur so that it is more attractive to cooperate rather than defect. Arguably this is a limited way of modeling the normative dimension of institutions, and to explain compliance when punishment is absent or very mild. Once again, this problem can be resolved by integrating equilibrium with rules accounts.

Rules make explicit the fact that social norms prescribe behavior and obligate people. Their basic structure is: In *S*, you ought to do *A.* However, the crucial question is how obligations might lead to action. For an obligation to motivate someone, she must regard the norm in which it features as valid. In other words, she must subscribe to that norm. This can be captured in terms of the notion of a normative belief, the belief that has the normative rule as its content. Note that, if it is a *social* norm, its very existence depends on other people, in particular on the beliefs or expectations they have. The idea that social norms can motivate can be captured positing preferences that are conditional on collective normative beliefs and expectations (Bicchieri, 2006; Hindriks, 2019, 2022a).^22^

To unify social theories is far from a trivial exercise, and comes with risks. Equilibrium theorists used to regard rules as redundant, and rule theorists objected to the rational choice framework the former relied upon. Thus, the attempt to unify these theories could easily have failed. In particular, we could have discovered that there is no way in which the notions of a rule and an equilibrium can coherently be combined in a single framework. Even so, this would have been rather surprising. After all, social scientists have corroborated both kind of theories. So, our main concern has been with how to make best use of them, to move from a partial to a more complete ontology.

### Social artefacts

Unified theories tend to be general and abstract, while folk ontology tends to focus on middle-sized, concrete objects and their everyday use. This raises the worry that unified theories end up being far removed from our ordinary understanding of the social world. To some extent, this is unavoidable: as we are interested in the big picture, social ontology must go beyond folk views and help us locate social entities in a wider context. But even so, a unified theory of institutions based on rules and equilibria can be used to develop an account of concrete objects that incorporates important insights from folk ontology.

Everyday life is inhabited by familiar objects such as police uniforms, traffic lights and wedding rings. Just as technical artefacts, such institutional objects are usually identified by their intended functions. More precisely, psychologists have established that the functions people rely on for the identification of artefacts are the ones that are *intended* by their *creators* (Bloom, 1996). Even though it may be used as a coat-hanger, a traffic light is a traffic light if it was originally built to regulate traffic.

Folk views provide a good starting point for understanding what a traffic light is, but they are also fallible, incomplete, and narrowly focused. In principle we would like to know what *kind* of objects traffic lights are, to have a theory that applies to objects of *that kind*, and that can be applied as widely as possible. Luckily, we do have a theory of this sort: it says that traffic lights are signaling devices, and it explains how such devices work *in general*. Such a theory helps locating such objects in a wider context.

**Fig. 1:**
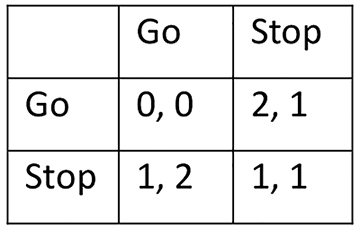
The crossroad (Hawk-Dove) game

To say that an object has a function is to say that it solves a problem of some sort. What kind of problems do traffic lights solve then? From an equilibrium perspective, the interaction that takes place at a crossroad is formally analogous to a game known as Hawk-Dove or Chicken (see Fig. 1). The *columns* of the matrix represent the actions that are available to drivers who travel along the north-south axis, and the *rows* represent the actions of drivers who travel east-west. Hawk-Dove is a game with two equilibria in pure strategies, (Stop, Go) and (Go, Stop). If the first driver decides to go, the wise strategy for the second one is to stop – and vice versa. Each one would prefer to go, so as not to waste time. But it would be disastrous if they both pressed ahead, and frustrating if they both stopped.

Institutions are systems of rules that help to solve problems of this kind, simply and smoothly. A simple rule of the road, for example, might give priority to the drivers who use the main road (the north-south motorway, say). A different rule may give priority to drivers who approach the crossroad from the right, regardless of where they are headed. And so on and so forth. But rules like these will typically privilege a set of drivers while discriminating others: the inhabitants of the western villages, for example, may have to spend a long time cueing at the crossroad, while the drivers from the northern city swiftly pass by.

**Fig. 2:**
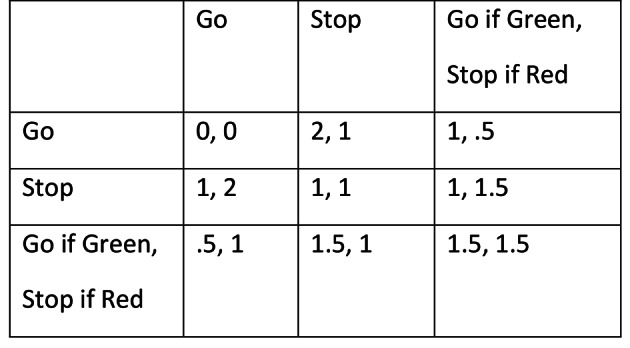
Augmented crossroad (Hawk-Dove) game

Introducing a traffic light, along with the corresponding rules, is one possible way to address issues of this kind. Traffic lights exploit a technology that sends intermittent signals, which can be observed publicly by all the drivers who approach a crossroad. The rules are familiar: ‘Go if green’, ‘Stop if red’. We can add these new strategies to the original matrix, enlarging the space of possible outcomes as in Fig. 2. The payoffs here have been calculated assuming that red and green signals are each sent 50% of the time, but other assumptions would work as well. The key point is that by augmenting the game in this fashion a new ‘correlated equilibrium’ comes into being (in the bottom-right corner, with payoffs 3/2, 3/2, in this particular case).^23^ Such an equilibrium differs significantly from the previous ones, because its payoffs are *symmetric*: over time, all drivers will spend an equal amount of time waiting at the crossroad, and feelings of injustice or discrimination are likely to subdue.

So far, the account of traffic lights as coordination devices might seem to be consonant with the folk view. But there is an important difference. The fact that an object is a coordination device can be the *unintended consequence* of individual actions. The theory of rules in equilibrium allows to explain also those cases in which a coordination function has not been (intentionally) imposed by any one at all. Consider, for instance, a physical barrier that for many years has impeded the movement of people across two territories (a river, for example). The river has worked, *de facto*, as a border. Now, the same entity may continue to function as a border after it ceases to constitute a physical barrier – if the river has dried up, for example – even if the people involved do not give it any thought (see Guala & Hindriks, 2015). What matters is the signaling function: if it’s north of the river, it’s my country; if it’s south of the river, it’s yours.

We take this to be a virtue of the unified theory. Good social ontology should have general scope and should be able to highlight similarities across cases that may appear heterogeneous to the untrained eye. So even though institutional objects, according to the folk, have intended functions, a proper (general, unifying) account of institutions should allow for other kinds of functions as well. As we shall see shortly, a simple distinction between *teleological* and *etiological* functions can carry us a long way (Hindriks, 2020; Hindriks & Guala, 2021). And it can also do other useful work in the philosophy of institutions.

## The significance of social ontology

### Ontology and amelioration

According to a venerable tradition that is deeply entrenched in Western philosophy, ontology is concerned with *what there is* and with its nature. Its main goal is to give us an accurate, general representation of the world. The distinction between descriptive and revisionary metaphysics (Strawson, 1959; Kriegel, 2013) falls in this tradition: revisionary accounts are supposed to provide a *more accurate* picture of the way the world is. Neither descriptive nor revisionary metaphysics are concerned with the way the world *should* or *ought* to be.

However, this traditional perspective has recently been questioned by philosophers who assign an overtly political or ethical mission to social ontology.^24^ Sally Haslanger, for example, presents her own ‘ameliorative project’ as follows:

In developing social constructionist accounts of race and gender, I’ve maintained that my goal is *not* to capture the ordinary meanings of ‘race’ or ‘man’ or ‘woman’, nor is it to capture our ordinary race and gender concepts. I’ve cast my inquiry as an analytical—or what I here call an *ameliorative*—project that seeks to identify what legitimate purposes we might have (if any) in categorizing people on the basis of race or gender, and to develop concepts that would help us achieve these ends. I believe that we should adopt a constructionist account not because it provides an analysis of our ordinary discourse, but because it offers numerous political and theoretical advantages. (2012, 366)

Philosophers like Haslanger are usually interested in the ontology of social kinds – such as gender or race – that play a particularly sensitive (and contested) role in our societies. They take an openly political stance that is critical with respect to ontology as it has traditionally been conceived of.

Folk views of the social world are imbued with normative significance: we cannot help but see the world as right/wrong, to compare what happens with what *should* happen, and so forth. The very language that we use reveals this attitude. It is perfectly natural to offer descriptions that simultaneously evaluate a social event, for example: ‘the bully stole the toy from the little boy’; ‘the corrupted civil servant took a bribe from a criminal’, and so on. These ‘thick’ descriptions convey information about the world as it is, about how it should be, and about what we should do to make it better.

Philosophical theories are bound to interact with the way in which sensitive categories like gender or race are used. And since social categories affect social behavior, ontological inquiries have a role to play in the maintenance or disruption of existing social structures. Haslanger’s project takes explicit responsibility for this. What she calls ‘ameliorative analysis’ is intended to lay bare societal problems and issue in concepts that are meant to have desirable consequences. To this end, she builds normative notions in her analyses of race and gender. For instance, she proposes that men are advantaged or privileged while women are disadvantaged or oppressed. She advocates that ontology be consciously guided by normative considerations. Thus, her ameliorative project revolves around politicized concepts.

The ‘ameliorative approach’ raises the question of how social ontology can contribute to social causes, such as social justice, and social change. It harbors a range of projects, which differ with respect to their inputs and outputs. Haslanger rejects folkism, as she does not regard the analysis of ordinary concepts as the primary goal of social ontology. But she seems to leave some role for the social sciences. They can play a role in what she calls ‘descriptive analysis’, which she regards as a prerequisite for ameliorative analysis (Haslanger, 2012). This suggests that, in addition to philosophy, the social sciences are inputs for her project. As mentioned, politicized concepts form the output.^25^

In what follows, we defend a version of the ameliorative approach that is based on our ecumenical methodology. The idea is that both folk ontology and scientific ontology can make significant contributions to it. In particular, social justice and social change stands to benefit from a solid understanding of the discrepancy between ideal and reality. Our proposal also differs with respect to outputs, as we do not require that amelioration is achieved by means of politicized concepts. To give content to this idea, we go on to present a functionalist framework that can be used to identify discrepancies between how things actually work and how they should work. This in turn provides a fruitful point of departure for promoting social justice and social change.

### Social functions and social change

Theories that combine equilibria with rules are suitable for evaluation, diagnosis and prescription, or so we propose. To begin with, equilibrium theories can illuminate why social change is difficult and often frustratingly slow (Valian, 1999). Social structures are self-reinforcing in a way that is neatly captured by the formal notion of an equilibrium. In equilibrium, people’s beliefs are not only true but also constantly confirmed by the actions of others. For these reasons, existing institutions tend to persist.

Perhaps more surprisingly, equilibrium theories can also help us envision better alternatives. To appreciate this, it is important to realize that such theories allow for the existence of suboptimal equilibria. It can also be that an equilibrium is approved by no one in a given society. And of course, a self-reinforcing institution can be morally despicable, for instance unjust or oppressive (e.g. Kuran, 1997, Chwe, 2013, Basu, 2018). In such situations, there may be another equilibrium that is better, in the sense of realizing values that the citizens actually endorse, or that they *should* endorse from the perspective of some ‘ideal’ theory. Thus, equilibrium theories are not limited to what is actual, but can also be used to explore what is possible.

Identifying a discrepancy between ideal and reality is the first step towards social change. Equilibrium theories are extremely useful for imagining alternatives to the status quo. However, evaluating them for ameliorative purposes requires normative judgments, typically ethical or political judgments about the way the social world ought to be. And such judgments are sometimes so disconnected from the way the world actually is that they are not conducive to social change.^26^ But a more basic problem is that a desirable alternative may be formulated in terms that are too far removed from the theory used to describe the status quo.

That’s why raising awareness by means of politicized concepts can only be part of the story. Ameliorative theory is not going to guide social change unless the preferences, beliefs and behaviors that go with it are self-fulfilling in the requisite way. More generally, philosophers’ interest in concepts is far from sufficient, and must be complemented by the best modelling tools – payoffs, beliefs, incentives – that contemporary social science provides. Thus, we propose that both rules and equilibria are crucial theoretical tools for ameliorative analysis and social change.

To analyze the relationship between values, rules, and equilibria, we propose a functionalist framework. The idea is that a problem can be diagnosed in terms of the function that an institution actually has and the function it should have. This proposal can be developed by distinguishing between ‘etiological’ and ‘teleological’ functions (Hindriks & Guala, 2021). An etiological function explains the existence and persistence of an entity. It does so in terms of the consequences that it gives rise to, which somehow reinforce the behavior that causes them. Teleological functions instead are assigned to an entity by purposeful agents such as human beings. Furthermore, they are normative. Teleological functions concern, for instance, the purpose that the entity should serve or the value that it realizes ideally. Thus, the distinction between etiological and teleological functions provides a bridge between the descriptive and the normative.

The notion of a teleological function is in turn closely related to that of a rule. Institutional rules play a vital role in almost any process of social change. They might simply reflect the incentives people have. But they can also imbue their actions with meaning. Furthermore, they are often supported by certain purposes that the participants of an institution want to achieve or by the values they take them to realize. Because of this, implementing a teleological function often requires altering the rules of an institution.

This functionalist framework reveals how important consilience can be for bridging the gap between descriptive and normative theorizing. On the one hand, the notions of a rule and a (teleological) function on which we rely are closely related to the corresponding common sense notions. Because of this, they are useful as input for policy or activism. On the other hand, equilibrium analysis can be used to delineate the limits and possibilities of social change. After all, a realistic alternative to the status quo must display the characteristics that make successful institutions stable and robust over time: they must be equilibria of the complex games that people play.

Thus, an ecumenical social ontology can contribute to social justice and social change. In this way, this section illuminates the broader significance of social ontology – and thereby answers our last question (Q3).

## Conclusion

Social ontology is an increasingly popular discipline, as is evidenced by this special issue. And for good reasons, we believe. It uncovers new puzzles of its own. And it sheds new light on existing puzzles in related disciplines that were, until recently, considered in abstraction from their social dimension. Instead of tackling directly the ‘what’ question, in this paper we have asked how social ontology is to be practiced, and for which purpose. As we see it, social ontology is a particularly promising discipline because of its potential to build bridges. We have proposed an ecumenical methodology by distinguishing three ways in which it can and should interact with other disciplines and perspectives. First, social ontology can forge connections between social scientific theories by unifying them. Second, it can preserve part of the manifest image by scrutinizing it from the perspective of the scientific image, integrating the two and thereby achieving consilience. Third, it can fruitfully interact with a range of philosophical disciplines that engage in normative theorizing. We hope that our bridge-builder conception of social ontology will contribute to an even more fecund way of practicing social ontology.

## Footnotes

1 One meaning of ‘consilience’ is that evidence from different sources supports the same conclusion. Another is that knowledge from different sources can be used to formulate a coherent and comprehensive theory across different sciences and possibly the humanities. We use the term in this second sense and include common sense as a possible source of knowledge.

2 In 1932 the British economist Lionel Robbins published *The Nature and Significance of Economic Science*, a little monograph that somehow managed to *describe* the method of economics, as well as to *prescribe* the way in which good economics should be done. We borrow part of Robbins’ title to signal (immodestly, perhaps) that we are trying to pull the same trick here.

3 Ecumenist projects, strictly speaking, do not always aim at synthesizing a plurality of options (say, religions), but sometimes aim at peaceful coexistence. As a reviewer pointed out, our goal of unification and consilience thus configures a particularly strong version of ecumenism.

4 Scientism is particularly popular in relation to the physical domain. Maudlin puts it as follows: ‘The basic idea is simple: metaphysics, insofar as it is concerned with the natural world, can do no better than to reflect on physics. Physical theories provide us with the best handle we have on what there is, and the philosopher’s proper task is the interpretation and elucidation of those theories’ (2007, 1). In a similar vein, Ladyman et al. argue: ‘The aim of this book is to defend … a metaphysics that is motivated exclusively by attempts to unify hypotheses and theories that are taken seriously by contemporary science. … [N]o alternative kind of metaphysics can be regarded as a legitimate part of our collective attempt to model the structure of objective reality’ (2007, 1).

5 That he is not the only one is suggested by Harold Kincaid’s claim that ‘much of the current social ontology literature is on my view largely an unfortunate and sad extension of unfruitful analytic metaphysics.’ (2015, 41).

6 Kriegel (2013) makes basically the same point in relation to what Strawson calls ‘descriptive metaphysics’, which is concerned with ‘the actual structure of our thought about the world’ (1959, 9). It explicates ‘what the structure of reality would be *if* it were accurately mirrored in the structure of our conceptual scheme, the conceptual scheme we actually have.’ (Kriegel, 2013, 1).

7 This line of argument is often used to reject challenges from experimental philosophy. Without delving into this debate, let us just say that we are sympathetic towards experimental philosophy, and more generally with those who try to collect systematic (rather than casual) evidence about concepts and their application.

8 For accounts of dependence and ‘grounding’ in social ontology, see Epstein (2015), Griffith (2018) and Schaffer (2019).

9 The only theory of marriage we could find that even mentions the term ‘ring’ is ‘ring cohesion theory’. But it studies networks or rings of marriages and how they integrate social groups (White, 2004).

10 If this is adopted as an important goal within the discipline, it may result in *different* consilient theories. And this is as it should be: competition between theories is likely to increase their quality. (As noticed by a reviewer, consilience may also *reduce* the number of competing theories. So, we take it to be a contingent matter.)

11 Since the seminal work of Bozzi (1958), Hayes (1979), a large body of experiments on folk physics has demonstrated that people make systematic mistakes in predicting and explaining relatively simple and familiar phenomena – the fall and acceleration of middle-sized objects in the vicinity of the Earth, for example. Research into folk social ontology has only recently begun (see Rose et al., 2018 and Noyes & Keil, 2019).

12 See Guala (2010, 2016) for criticisms of Ruben (1989) and Thomasson (2003).

13 We discovered this city regulation from Austro-Hungarian times, which was never repealed, on https://www.thelawyerportal.com/blog/top-10-weirdest-laws-around-world/.

14 Baker’s (2007) approach to social ontology can also be characterized as descriptive metaphysics (see note 6).

15 Kincaid also argues that ‘if there is value to social ontology, it has to be in contributing to empirical social science’ (2021, 41). Elsewhere, he supports inference to the best explanation, which suggests that he also believes that the social sciences can contribute to social ontology (Kincaid, 1997). Just as Kincaid, Lauer (2019), Saunders (2020), Little (2020), and Lohse (2020) can plausibly be regarded as dualists, even if they differ in the faith they put in the social sciences.

16 Pettit (1995, 2000) proposes to reconcile rational choice theory with common sense. As such, his methodology can also be characterized as ecumenical. Hindriks (2021, 2022b) provides critical discussions that clarify similarities and differences with respect to our approach.

17 Dupré (1993), Galison & Stump (1996), and Cartwright (1999) extensively discuss the lack of coherence within the sciences. They conclude from this that reality is disunified too. Although we endorse the same premise, we resist this conclusion: different theories and models are best suited to explain different phenomena, but a full understanding of the way the world is calls for an explanation of how different domains or aspects of reality are *related*.

18 See Kincaid (1997) for a discussion of interlevel unification, or integration of social scientific theories with sciences that concern lower levels, such as biology. He regards this as an empirical rather than a conceptual issue. In contrast, we believe that it is partly a conceptual issue, in particular insofar as dependence relations are concerned.

19 Our position on unification as a goal of ontology is thus closer to that of Ladyman et al. (2007), then to the radically ‘disunified ontology’ of Dupré (1993) and Cartwright (1999).

20 Devices such as verbal instructions, traffic lights, or even physical traits can represent symbolically and are assigned meanings by their users.

21 This rule applies in situation *S*, which can be made explicit as follows: In *S* (If *D*, do *A*).

22 An interesting implication of this approach is that a norm can increase people’s motivation even if it does not actually achieve full cooperation. So, the notion of norm-governance connects rules to equilibria without gluing them together. This is why this second theory is called ‘the Rules-*and*-Equilibria Theory’ (see Hindriks 2019, 2021, 2022a).

23 The notion of correlated equilibrium was introduced by Aumann (1974) and applied to social conventions by Vanderschraaf (1995).

24 See e.g. Mallon (2006), Haslanger (2012), Barnes (2014), Diaz-Leon (2013), Mikkola (2016), Asta (2018).

25 Hawley (2018) is rather skeptical about the idea that the social sciences can be used as input for social ontology (Sect. 1.3). At the same time, she embraces Haslanger’s ameliorative project. This suggests that she regards it as a purely philosophical project. Strikingly, given how poorly she rates them, Hawley argues that the social sciences can contribute to the ameliorative project in a number of ways. For one thing, they can be useful given that ‘we need to understand our goals, and how to achieve them’. She adds that ‘both of these tasks require empirical input alongside armchair reflection’ (2018, 196) Furthermore, the social sciences can be useful for calculating the costs and benefits of certain interventions, as well as their consequences. Finally, they can help to identify which politicized concepts serve our legitimate purposes best. In a sense, the social sciences perform the role of the underlaborer, in the ameliorative approach as Hawley conceives of it.

26 This is a common objection against ideal theory in political philosophy (Sen, 2009).
